# A Lesson from Plants: High‐Speed Soft Robotic Actuators

**DOI:** 10.1002/advs.201903391

**Published:** 2020-01-21

**Authors:** Richard Baumgartner, Alexander Kogler, Josef M. Stadlbauer, Choon Chiang Foo, Rainer Kaltseis, Melanie Baumgartner, Guoyong Mao, Christoph Keplinger, Soo Jin Adrian Koh, Nikita Arnold, Zhigang Suo, Martin Kaltenbrunner, Siegfried Bauer

**Affiliations:** ^1^ Soft Matter Physics Institute of Experimental Physics Johannes Kepler University Linz Altenberger Straße 69 Linz 4040 Austria; ^2^ Soft Materials Lab Linz Institute of Technology LIT Johannes Kepler University Linz Altenberger Straße 69 Linz 4040 Austria; ^3^ Institute of High Performance Computing A*STAR 1 Fusionopolis Way, #16‐16 Connexis Singapore 138632 Singapore; ^4^ Institute of Polymer Science Johannes Kepler University Linz Altenberger Straße 69 Linz 4040 Austria; ^5^ Department of Mechanical Engineering University of Colorado Boulder Boulder CO 80309 USA; ^6^ Materials Science and Engineering Program University of Colorado Boulder Boulder CO 80303 USA; ^7^ Department of Mechanical Engineering National University of Singapore Singapore 117575 Singapore; ^8^ John A Paulson School of Engineering and Applied Sciences Harvard University 29 Oxford Street Cambridge MA 02138 USA

**Keywords:** bioinspired dielectric elastomer actuators, coupled dielectric elastomer balloons, snap‐buckling, snap‐through instabilities, soft robotics for high‐speed actuation

## Abstract

Rapid energy‐efficient movements are one of nature's greatest developments. Mechanisms like snap‐buckling allow plants like the Venus flytrap to close the terminal lobes of their leaves at barely perceptible speed. Here, a soft balloon actuator is presented, which is inspired by such mechanical instabilities and creates safe, giant, and fast deformations. The basic design comprises two inflated elastomer membranes pneumatically coupled by a pressurized chamber of suitable volume. The high‐speed actuation of a rubber balloon in a state close to the verge of mechanical instability is remotely triggered by a voltage‐controlled dielectric elastomer membrane. This method spatially separates electrically active and passive parts, and thereby averts electrical breakdown resulting from the drastic thinning of an electroactive membrane during large expansion. Bistable operation with small and large volumes of the rubber balloon is demonstrated, achieving large volume changes of 1398% and a high‐speed area change rate of 2600 cm^2^ s^−1^. The presented combination of fast response time with large deformation and safe handling are central aspects for a new generation of soft bio‐inspired robots and can help pave the way for applications ranging from haptic displays to soft grippers and high‐speed sorting machines.

## Graphical abstract

A voltage‐triggered soft balloon actuator with an impressive displacement (1398% total volume change) at high speed (2600 cm^2^ s^−1^ area change rate) is developed by harnessing the mechanical snap‐through and snap‐back instability of a rubber balloon. The trigger actuator is pneumatically coupled to the high‐speed actuator. This concept promises applications in soft bio‐inspired systems in modern robotics and engineering.

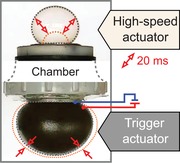

## 1 Introduction

**Figure 1:**
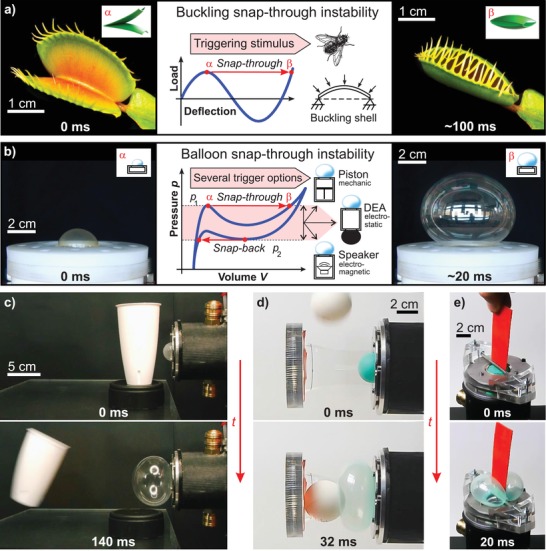
Harnessing mechanical instability to improve the speed of actuation. a) The Venus flytrap uses a stimulus‐triggered mechanical buckling instability. b) Mechanical balloon snap‐through instability enables high‐speed actuation. Characteristic pressure–volume behavior of a rubber membrane: As soon as the pressure reaches the critical pressures *p*_1_ or *p*_2_, a further insignificant pressure increase or decrease rapidly changes the membrane volume in just a few milliseconds. Under pressure‐controlled conditions, snap‐through instability can be triggered by different kinds of pressure sources. c) Possible application as fast sorting device, e.g., for conveyor belts; d) fast and soft gripper catching a ping‐pong ball; e) handling of sensitive objects by improving compliance with additional constraints (Video S1, Supporting Information).

Nature inspires artificial systems, particularly regarding motion. Quick and large movements are keenly sought‐after for applications in rigid and soft robotics, industrial automation, and modern prosthetics.^1^, ^2^, ^3^, ^4^, ^5^ In recent years, the development of artificial muscles that mimic the basic function of human and animal musculature has gained an importance due to its wide range of potential applications.^6^ But there are examples in which direct muscle action alone cannot be responsible for the rapid movement. The jaw muscles of hummingbirds are not strong enough to close the beak in the observed short amount of time. However, hummingbirds are able to bend their lower jaw and use a controlled elastic instability to rapidly snap it from the open to the closed position.^7^ Completely without muscle support, plants rely solely on mechanical instabilities for rapid movement. The inherent motility of plants is the consequence of selective swelling and shrinking caused by water flow driven by osmosis and evaporation phenomena.^8^ These processes are rather slow and therefore limit the overall speed. Plants like the Venus flytrap can overcome this limit by suddenly releasing stored elastic energy resulting in one of the fastest movements (≈100 ms) in the plant world.^9^, ^10^ The rapid closure of the Venus flytrap is made possible by harnessing a snap‐buckling instability (**Figure** **1**a). Due to geometric constraint and elastic properties of the doubly curved terminal lobes which form the trap at the tip of each leaf the plant can accumulate elastic energy and release it if triggered by an external stimulus.^9^, ^11^ Here, we took a lesson from plants and transferred the idea of how mechanical instabilities are key to the rapid actuation to technical applications. By exploiting an elastic snap‐through and snap‐back instability, we show that the speed of elastomer balloon actuators can be accelerated drastically. This possibility results from the nonmonotonic N‐shaped pressure–volume relation of an inflated rubber membrane.^12^, ^13^, ^14^ As soon as the pressure reaches the critical values *p*_1_ or *p*_2_, a further insignificant pressure increase or decrease rapidly changes the membrane volume within milliseconds (Figure 1b). Pressure‐controlled deformation is required for the occurrence of an instability,^15^, ^16^ therefore the rubber membrane is mounted on a pressure vessel of suitable size. The pressure signal triggering the balloon instability can be provided by any kind of controllable pressure source like a pneumatic compressor, a loudspeaker, or a coupled dielectric elastomer actuator (DEA; Figure 1b). We chose an electroactive acrylic elastomer (3M VHB 4910F) as actuator material to electrically manipulate the pressure of the system.^17^, ^18^ VHB has been widely employed due to its large stretchability and high electric breakdown strength.^16^ With voltage‐triggered balloon instability there is no need for fast and complex pneumatic pressure equipment, after initially pressurizing the chamber. Actuators based on soft balloons are compliant, robust, light weight, simple in structure, and have low costs. Considering these desirable features, they are widely used, ranging from pneumatic applications in medical and health‐care robotics to wave‐handling systems to transport delicate objects in industry.^19^, ^20^, ^21^ All the mentioned examples would benefit from the increased response speed. To demonstrate the underlying potential, we present prototypes utilizing the snap‐through instability of a balloon. In Figure 1c, a fast sorting device is shown, which can be used to sort out defective parts on a conveyor belt. A gripper as depicted in Figure 1d, is fast enough to reliably catch falling objects of various shapes. More controllable gripping can be provided by an appropriate geometric design. A simple example is shown in Figure 1e, where a mounted rod blocks the balloon, resulting in better compliance to the object's shape and thus ensures safe handling.

Except for lab demonstrations, DEAs solely based on VHB acrylic adhesives—favored for achieving giant static strain—have very limited potential for commercial applications that require fast actuation due to viscoelasticity.^22^ We overcome this problem by pneumatically coupling two balloon actuators of dissimilar materials. In our approach, a VHB DEA triggers the instability of an inflated natural rubber membrane, which serves as high‐speed balloon actuator. Therefore, we are able to report giant deformations within 20 ms. The properties of natural rubber enable high‐speed volume expansion rates up to 2300 cm^3^ s^−1^ (2600 cm^2^ s^−1^ area expansion rate) at forward and backward actuation at frequencies up to 8 Hz. We ensure safe operation, as there is no high voltage at the high‐speed balloon actuator. At the same time, the trigger actuator (TA) undergoes small deformations only, averting failure mechanisms such as electromechanical instability or electrical breakdown (EB).^23^ The actuation is remote, potentially allowing significant spatial separation into a passive and an electrically active part, which can be arranged “behind the scenes.” Subsequent experiments and theory demonstrate how the rubber balloon achieves high‐speed and giant deformation by harnessing the snap‐through and snap‐back instability. The analysis is based on an electromechanical model using hyperelasticity of elastomer membranes.

## 2 Results and Discussion

### 2.1 Experimental Setup and Operation Principle

**Figure 2:**
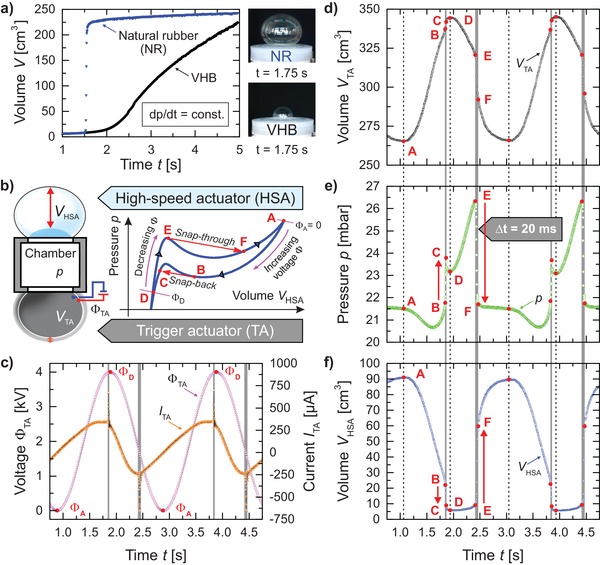
a) Comparison of the volume expansion rate when using elastomer membranes of low viscosity (natural rubber) and high viscosity (acrylic elastomer VHB 3M), while undergoing pressure‐controlled mechanical balloon snap‐through instability (Video S2, Supporting Information). b) Setup of a HSA remotely triggered by a coupled DEA balloon (TA) and schematic representation of a complete actuation cycle of the HSA in the pressure–volume plane. Giant volume changes occur rapidly as the HSA undergoes snap‐back (state B to C) or snap‐through (state E to F) instability. c) Sinusoidal voltage applied to the TA is used to trigger instability of a coupled balloon actuator. d) The volume change of an electrically driven DEA balloon actuator (TA) modulates e) the system pressure, f) enabling the coupled HSA to undergo instability with large volume changes.

We build our system of coupled balloons by mounting a dielectric elastomer membrane (VHB) as TA and a rubber balloon featuring low viscoelasticity as high‐speed actuator (HSA) on a chamber of suitable volume. In a previous experiment with VHB elastomers, the snap‐through occurred on a time scale of 100 s, mainly due to the large viscoelasticity of the VHB elastomer.^15^ Thus, VHB is not suitable for fast snap‐through. Natural rubber, on the other hand, has comparatively smaller viscoelasticity, enabling large volume changes over a short time. **Figure** **2**a compares the “creep‐through” of the VHB membrane with a fast snap‐through of the rubber membrane. The TA is constantly connected to a high voltage supply providing the voltage signal Φ_TA_ (Figure 2b). The schematic in Figure 2b shows the cyclic process in the pressure–volume plane of the HSA (*p*‐*V*_HSA_). Each pressure–volume state of the HSA corresponds to a voltage Φ_TA_ applied to the coupled TA. The operation of the HSA is separated into the following steps: The initial pressure of the system is set to *p*_A_ of state A, slightly above the verge of instability of the rubber balloon in state E to enable electrically triggered, giant deformation. Then the voltage Φ_TA_ is applied to the TA membrane. It becomes thinner, volume *V*_TA_ increases, and the common pressure in the systems falls, consequently decreasing the volume *V*_HSA_ of the HSA. As soon as the pressure in the system falls below *p*_B_, the HSA snaps back from state B to C. At state D, the voltage Φ_TA_ and volume *V*_TA_ reach their maximum and *V*_HSA_ its minimum. Subsequent reduction of the voltage at the TA leads the system to the verge of instability in state E at pressure *p*_E_. The instability is triggered and the HSA snaps through to state F and finally returns to state A when the voltage at the TA reaches its minimum. The unstable states B and E are characterized by the pressure–volume curve of the HSA being tangent to the pressure–volume curve of the air in the whole system.^15^ This is the first demonstration of harnessing an electrically triggered snap‐*back* instability of a balloon actuator—a key element to achieve fast *cyclic* actuation.

### 2.2 Discussion of Measured Data

In our experiment, we measured the voltage Φ_TA_ and current *I*_TA_ of the TA as well as the system overpressure *p* and determined the volumes *V*_HSA_ and *V*_TA_ of the TA and HSA for several cycles of operation. Despite the first two consecutive measurements being noticeably different, the behavior became reproducible after three to four cycles. Figure 2c–f illustrates the measurement data for two consecutive cycles.

**Figure 3:**
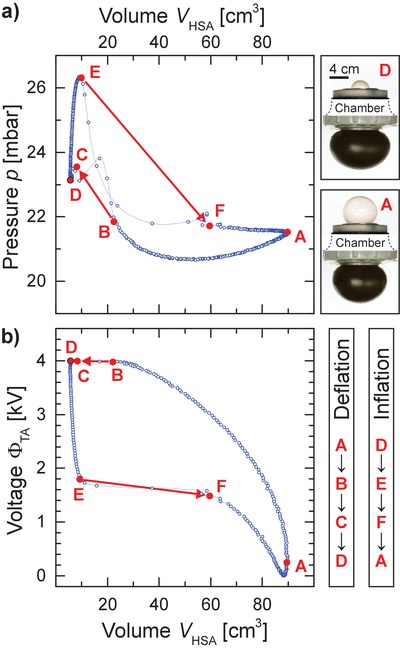
a) Measured pressure and volume data of the cyclic experiment. The system is pressurized to an initial pressure *p*_A_. As voltage is applied to the TA, the change of volume and pressure forces the coupled HSA to undergo the snap‐through and snap‐back instability (indicated by arrows). The characteristic states A to F of a full actuation cycle correspond to the points marked in Figure 2b. The shapes of the balloon at state A and D of the TA and HSA are depicted for comparison. b) Measured voltage Φ_TA_ applied on the TA plotted as a function of the volume *V*_HSA_ of the HSA.

By applying sinusoidal voltage (@ 0.5 Hz and 4 kV peak‐to‐peak) to the compliant electrodes, the TA deforms cyclically (Figure 2c,d and Video S3, Supporting Information). Similar to a pumping piston, the alternating pressure caused by the TA membrane triggers the snap‐through and snap‐back instability of the HSA (Figure 2e,f and Video S3, Supporting Information). A suitable prestretch of the TA is necessary to obtain sufficient actuation,^24^ required to reach the unstable states B and E of the HSA. We observe that the sudden changes of volume and pressure during the instabilities from state B to C, or from state E to F result in distinct spikes in the current characteristics *I*_TA_ (orange triangles with black connecting lines in Figure 2c). They originate from the rapid alteration of the capacitance of the electroactive membrane due to the variation in geometry (area and thickness) during inflation or deflation. The observed small currents during operation permit the usage of cheap commercially available low power DC to HV converters for high voltage supply. The time *t*_EF_ for the HSA to change from state E with volume *V*_HSA,E_ = 13 cm^3^ to state F with *V*_HSA,F_ = 59 cm^3^ is 20 ms. This results in a high volume expansion rate of 2300 cm^3^ s^−1^ (2600 cm^2^ s^−1^ area expansion rate in spherical membrane approximation). The total volume change of one half‐cycle between state A at volume *V*_HSA,A_ = 88.4 cm^3^ and state D with *V*_HSA,D_ = 5.9 cm^3^ is about 1398%. The snap‐through from state E to F results in a significant pressure drop and a rapid volume increase. During a snap‐back instability from state B to state C, the pressure rises rapidly and the volume drops quickly. The size of these jumps can be modified by changing the volume of the connecting chamber.^15^, ^16^ For subsequent theoretical analysis, the measured cyclic time‐dependent data sets are plotted parametrically in the pressure–volume plane for the HSA, as well as the voltage applied on the TA versus the volume of the HSA (**Figure** **3**).

### 2.3 Theoretical Analysis

The resulting blue pressure curve in Figure 3a shows the typical hysteretic behavior of elastomer balloons.^25^ This hysteresis is due to stretch‐induced crystallization (SIC) and viscoelastic effects. Its theoretical analysis is possible within the frame of microscopic theories^26^, ^27^ and quasi‐linear viscoelasticity,^28^ but is not instructive for our purposes. The hysteresis in the pressure–volume plane of the HSA does not change the qualitative picture, though it does influence the exact numbers. We account for this effect by using different shear modulus μ_HSA_ and *J*_lim_ for the inflation and deflation stages (Table S1 and Figure S1a, Supporting Information).

For theoretical analysis, the system is idealized by assuming the HSA and TA to be spherical with initial (unstretched) radii *R*_HSA_ and *R*_TA_ and thicknesses *H*_HSA_ and *H*_TA_. The membranes of both balloons are taken to be incompressible and thin. When the pressure inside each balloon exceeds the atmospheric pressure *p*_atm_ by *p*, the balloons deform to radii *r*_HSA_ = λ_HSA_ *R*_HSA_ and *r*_TA_ = λ_TA_ *R*_TA_, where λ are the respective homogenous radial (lateral) stretches. In addition to pressure, the TA is subjected to the voltage Φ_TA_. We consider quasi‐static equilibrium with respect to pressure and voltage. We assume that air is an ideal gas obeying the ideal gas law

(1) $$\left(p+p_{\text{atm}}\right)\;\left(\frac{4}{3}\pi R_{\text{TA}}^{3}\lambda_{\text{TA}}^{3}\;\;+\;\;\frac{4}{3}\pi R_{\text{HSA}}^{3}\lambda_{\text{HSA}}^{3}\;\;+\;\;V_{\text{C}}\right)\;\;=\;\;Nk_{\text{B}}T$$

here, *V*_C_ is the chamber volume, *N* is the number of air molecules, and *k*_B_ *T* is the temperature in energy units. The amount of air *N* enclosed by both balloons and chamber is fixed after the valve is closed. The deformation is assumed to be isothermal. In practice, the fast expansion and contraction of the considered prototype actuators may be closer to adiabatic processes, leading to additional effects, which will be discussed elsewhere. The adiabatic relative temperature changes, and the concomitant changes in the shear modulus Δμ/μ = Δ*T*/*T* can be estimated to be less than 1%. Such minor variations are below the accuracy of the current modeling.

We account for the stiffening of the elastomer at large deformation by using a hyperelastic Gent material model^29^ for the elasticity of both balloons, such that the volumetric strain energy density for equi‐biaxial deformation (configurational part of Helmholtz free energy) is of the form

(2) $$W_{\text{stretch}}\;\left(\lambda \right)\;\;=\;\;-\frac{\mu \;J_{\text{lim}}}{2}\ln\left(1-\frac{2\lambda^{2}+\lambda^{-4}-3}{J_{\text{lim}}}\right)$$

here, *μ* is the small‐strain shear modulus and *J*_lim_ is a constant related to the stiffening of the elastomer at large deformation.^29^, ^30^ For the dielectric elastomer balloon, we adopt the model of ideal dielectric elastomers,^31^ such that the free energy density is the sum of two parts: the elastic energy due to stretching in Equation (2) and the electrostatic energy density due to the polarization of the elastomer, *W*_ele_ = *D* ^2^ / (2ε), where *D* is the electric displacement and ε is the absolute permittivity.

For any variation of the system, the free energy of each balloon membrane varies by its respective 4*πR* ^2^ *H δW*. When the charge on the electrodes of the TA varies by *δQ*, the applied voltage does work Φ_TA_ *δQ*. When the radius of either balloon varies by *δr*, the pressure does work 4*πr* ^2^ *p δr*. For the HSA, a state of equilibrium is reached when the variation of the free energy of the membrane equals to the work done by the pressure; in the case of the TA, the equilibrium state is reached, when the variation of the free energy of the membrane is equal to the combined work done by the pressure and the voltage. This results in standard N‐shaped single‐balloon pressure–volume dependences *p*_HSA_(*V*_HSA_) and *p*_TA_(*V*_TA_, Φ_TA_), as detailed in the Supporting Information (Equation (S2)). They correspond to the equilibrium conditions of the kinematic set of equations derived previously.^32^ Fitting of the pressure–volume data for the rubber (HSA) and VHB (TA) membranes (Figure S1, Supporting Information) results in the values (Table S1, Supporting Information) used in the calculations. They comply with the data reported and used in literature.^16^ The relative permittivity ε_r_ of VHB is taken as 4.7.^33^

**Figure 4:**
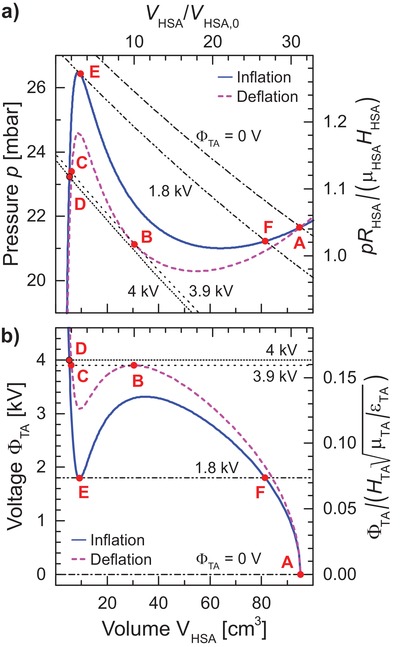
Theoretical a) static pressure *p* and b) voltage Φ_TA_ applied to the TA membrane as a function of the volume *V*_HSA_ of the HSA with dimensional left‐bottom axes, and dimensionless right‐top scales (with inflation *μ*_HSA_ value). The solid blue and the dashed pink curves represent the inflation and deflation stages and account for the material hysteresis observed in the experiment. Different dotted black curves combine *p*_TA_(*V*_TA_) dependences with air conservation (Equation (1)) for different voltages Φ_TA_, applied to the coupled TA: 0 V, the snap‐through, the snap‐back, and the maximum value (see Equation (S5), Supporting Information). The equilibrium pressure–volume states of the HSA for these voltages are marked by red dots and correspond to states indicated in Figures 2 and 3. Parameters are listed in Table S1 in the Supporting Information.

The cyclic dynamic behavior shown in Figure 3a,b is modeled in **Figure** **4**a,b, with the same notations for the key points. The solid blue curve and the dashed pink curve in Figure 4a do not depend on the TA and visualize the *p*_HSA_(*V*_HSA_) dependences. They correspond to the inflation (solid blue) and deflation (dashed pink) stage—deflation has a smaller μ_HSA_ and *J*_lim_ value. The *snapping* hysteresis exists also with constant parameters, but the numbers differ significantly. The different black curves combine the *p*_TA_(*V*_TA_, Φ_TA_) dependence with Equation (1), and do not depend on the HSA (see Equation (S5) in the Supporting Information for details). They correspond to 0 V, snap‐through, snap‐back, and the maximum value of the voltage. The parameters of the system are chosen such that the TA is always strongly stretched, and the applied voltage modulates its pressure. The equilibrium states are the intersections of the blue—or pink—and black curves. Snapping happens, when some of the common solutions disappear, i.e., when these curves become tangential. The snapping path roughly follows (dynamically modified) HSA single‐balloon curves. The *p*_HSA_(*V*_HSA_) dependence dictates a large pressure drop with a large volume expansion during snap‐through instability, and a smaller pressure rise with a smaller volume contraction during a snap‐back. The snapping magnitude is governed by the HSA properties, for snap‐through $\;\Delta p\leq {\left(C\frac{\mu H}{R}\right)}_{\text{HSA}}$ and $\frac{V_{\text{HSA},\text{after}}}{V_{\text{HSA},\text{before}}}\geq \frac{\lambda_{\text{lim}}^{3}}{7^{1/2}\;\;\times \;\;3^{3/2}}\;$ (see Equations (S8)–(S10) and S14, Supporting Information). Here, *C* is a constant $C\approx 12\times 7^{-7/6}-3^{3/2}\lambda_{\lim}^{-1}\approx 0.23$ containing the reciprocal of λ_lim_ which is the maximal possible (equal‐biaxial) stretch of the HSA according to the Gent model $2\lambda_{\text{lim}}^{2}\approx J_{\text{lim}}$. Without the effects of SIC, the hysteresis in the pressure–volume plane of the HSA vanishes and the blue and pink curves merge, but clearly, the *voltage‐induced snapping* hysteresis still persists, though the required voltage difference is significantly lower. Theoretical Figure 4b explains the dynamic voltage behavior, observed in the experimental Figure 3b; it is plotted according to the parametric procedure described in the Supporting Information. The voltages required for snapping depend on the HSA and TA properties and are of the order of $\Phi_{\text{TA}}\approx {\left(\frac{HR}{2\varepsilon \lambda_{\text{lim}}}\right)}_{\text{TA}}^{1/2}{\left(C\frac{\mu H}{R}\right)}_{\text{HSA}}^{1/2}$ (Equations (S14) and (S15), Supporting Information). The voltages only weakly depend on the shear modulus of the VHB, μ_TA_, because the change in the elastic pressure of the HSA between the snapping points is compensated largely by the changes in the electrostatic pressure of the strongly stretched TA. The theoretical predictions of Figure 4 are in a *semi‐quantitative* agreement with the experimental results from Figure 3. The details of the theoretical analysis and the influence of various parameters are described in depth in the Supporting Information.

With this good agreement between our model and our experiment, an optimized system that maximizes actuation performance can be designed in the future.

## 3 Conclusion

Inspired by nature, we introduced an actuator, which harnesses a snap‐through and snap‐back instability for giant high‐speed deformations. By using a system of coupled balloons, remote, high‐voltage triggered, bistable actuation is possible. Consisting of an electrically active and passive part, this actuator can be operated in a safe regime, far away from the EB voltage of the dielectric elastomer. Its combination of fast volume change rate and large maximum deformation makes this concept an attractive candidate for use in soft robotics.

## 4 Experimental Section

### Membrane Material and Preparation

The membrane of the HSA was made of high‐quality natural rubber Durex Ultra with a diameter of 32 mm and a thickness of 50 μm without prestretch. High‐quality rubber improved the reliability and speed of the snap‐through instability. The DE material used for the TA was the acrylic elastomer 3M VHB 4910F with a diameter of 45 mm and prestretch λ_pre_ = 1.8, coated on both sides with compliant electrodes. A stretchable electrode was prepared by coating the DE membrane with carbon grease (MG chemicals 846‐80G diluted with ELBESIL B50 silicon oil).^34^ The membrane geometries and mounting holes for the screws to fixate the membranes were cut out using a laser cutter (Trotec Speedy300).

### Fabrication of the Setup

Both membranes were mounted on a cylindrical chamber with a volume of *V*_C_ = 7 dm^3^ and clamped with rigid rings. A valve for the supply with pressurized air and the pressure sensor was directly connected to the chamber.

### Measurement Equipment

System‐pressure *p* was measured by a Jumo p30 pressure sensor and the volumes *V*_TA_ and *V*_HSA_ of both membranes were obtained by video analysis and contour detection using the image processing library National Instruments IMAQ. The DEA was connected to a Trek Model 610D high‐voltage power supply combined with a function generator (Hewlett Packard 33120A) to achieve a sinusoidal waveform. A DAQ‐card (National Instruments PCI‐6281) was used for data acquisition.

## Conflict of Interest

The authors declare no conflict of interest.

## Supporting information

Supporting Information

Supplemental Movie 1

Supplemental Movie 2

Supplemental Movie 3
