# Supplementary material for: A Lesson from Plants: High‐Speed Soft Robotic Actuators
Source: Adv Sci (Weinh). 2020 Jan 21;7(5):1903391. doi: 10.1002/advs.201903391 (PMC7055565; doi:10.1002/advs.201903391)
Supplement: Supplementary file 1 — Supporting Information [file ADVS-7-1903391-s001.pdf]

Copyright WILEY-VCH Verlag GmbH & Co. KGaA, 69469 Weinheim, Germany, 2020.

ADVANCED SCIENCE
Open Access

Supporting Information

for *Adv. Sci.,* DOI: 10.1002/advs.201903391

A Lesson from Plants: High-Speed Soft Robotic Actuators

Richard Baumgartner, Alexander Kogler, Josef M. Stadlbauer, Choon Chiang Foo, Rainer Kaltseis, Melanie Baumgartner, Guoyong Mao, Christoph Keplinger, Soo Jin Adrian Koh, Nikita Arnold, Zhigang Suo, Martin Kaltenbrunner, and Siegfried Bauer*

Copyright WILEY-VCH Verlag GmbH & Co. KGaA, 69469 Weinheim, Germany, 2018.

Supporting Information

A Lesson from Plants: High-Speed Soft Robotic Actuators

Richard Baumgartner, Alexander Kogler, Josef M. Stadlbauer, Choon Chiang Foo, Rainer Kaltseis, Melanie Baumgartner, Guoyong Mao, Christoph Keplinger, Soo Jin Adrian Koh, Nikita Arnold, Zhigang Suo, Martin Kaltenbrunner, and Siegfried Bauer*

HSA and TA balloons with connecting chamber

The HSA and TA balloons, together with the connecting chamber C, contain a *constant* amount of gas molecules *N*, which obey the ideal gas law:

$$(p_{atm}+p)(V_{HSA}+V_{TA}+V_C)=NkT \tag{S1}$$

Here, the corresponding volumes *V* are labeled by subscripts. Assuming *isothermal* sealed equilibrium conditions, both sides of this equality remain *constant.*

The equilibrium *over*pressure p inside the *thin spherical* balloon made of an *incompressible elastomer* can be expressed as follows:[12,35-37]

$$\begin{aligned} p &\overset{1}{=}\frac{H}{R}\frac{W_{str,\lambda}}{\lambda^2}\overset{2}{=}\frac{H}{R}\underset{J_\lambda/\lambda^2}{W_{str,J}\,4(\lambda^{-1}-\lambda^{-7})}\overset{3}{=}\frac{H}{R}\frac{2\mu(\lambda^{-1}-\lambda^{-7})}{1-(2\lambda^2+\lambda^{-4}-3)/J_{\lim}}\quad \text{a} \\ p &\overset{4}{=}\frac{H}{R}\frac{(W_{str,\lambda}-W_{ele,\lambda})}{\lambda^2}\overset{5}{=}\frac{H}{R}\frac{2\mu(\lambda^{-1}-\lambda^{-7})}{1-(2\lambda^2+\lambda^{-4}-3)/J_{\lim}}-\frac{2\varepsilon\Phi^2\lambda}{RH}\overset{6}{=}p_{str}+p_{ele} \end{aligned} \tag{S2}$$

Here, *H* and *R* are the thickness and the radius of an unstretched balloon, λ is the *radial* stretch, W_{str} is the *configurational* part of the volumetric energy density and $W_{str,\lambda}$ denotes its derivative with respect to λ. The expression $S2_1$ (labeled by 1 over the equality in Equation S2) represents the most general form, where W_{str} is expressed in terms of a single stretch variable λ assuming incompressibility and equal-biaxial stretch conditions. The equality $S2_2$ assumes that W_{str} depends only on the 1st stretch invariant *J*, which implies (under the same conditions):

$$W_{str} = W_{str}(J),\; J = \sum_{i=1}^{3} \lambda_i^2 - 3 = 2\lambda^2 + \lambda^{-4} - 3,\; J_\lambda = 4(\lambda - \lambda^{-5}) \qquad \text{(S3)}$$

J_λ denotes the derivative of the 1st stretch invariant J with respect to λ. Finally, the equality S2$_3$ is based on the (isothermal) Gent model (Equation 2, Main Text) with shear modulus μ and limiting stretch invariant value $J_{\lim}$. For this model, the energy derivative with respect to the invariant J has the simple form: $W_{str,J} = \dfrac{\mu/2}{1 - J/J_{\lim}}$. More complex material models should use the representation S2$_1$. Formulas S2$_{1\text{-}3}$ apply to the HSA balloon, for which the electrostatic energy does not have to be taken into account. Equation S2$_3$ can be used to estimate the stored energy in the HSA by calculating the integral under the $p_{HSA}(V_{HSA})$ curve in Figure 3a or 4a. The stored energy can also be determined by using the elastic energy density in Equation 2 with an elastomer volume of $V_{el} = (4\pi R^2 H)_{HSA} \approx 0.05\,\text{cm}^3$ (values from Table S1). Both methods yield the same result for the energy density of 4 J/cm^3 and an energy of 0.2 J for the maximum extension in state A (Figure 4a).

The pressure p in the TA balloon obeys similar mathematical expressions, when the contribution of the electrostatic energy W_{ele} is considered together with W_{str}. In SI units, the electrostatic energy density is:

$$W_{ele} = \frac{\varepsilon E^2}{2} = \frac{\varepsilon \Phi^2}{2h^2} = \frac{\varepsilon \Phi^2 \lambda^4}{2H^2} \qquad \text{(S4)}$$

Here, ε is the *full* dimensional dielectric constant that includes vacuum permittivity ε_0. The last equality implies $h = H/\lambda^2$ due to incompressibility. When the TA actuator is kept at a *constant voltage*, the work done by the battery *reverses the sign* of the electrostatic contribution.[31,38] This results in the expressions S2$_{4\text{-}5}$ for the pressure where W_{ele} enters with a negative sign. In the expression S2$_6$ we subdivide the overpressure p in the TA into stretching and (negative) electrostatic contributions, which we use later.

Equation S1 and S2 are *similar* to the *equilibrium* conditions discussed in the references.[15,16,32,39] However, these papers describe only one balloon (with or without a connecting chamber), some employ Neo-Hookean or Mooney-Rivlin material models, concentrate on the deviations from the spherical shape (studied numerically), influence of the pre-stretch, *dynamic* behavior, etc.

Quasi-static equilibrium analysis

Equation S1 and $S2_{3,5}$ (for the Gent models of the HSA and the TA) provide three equations for three unknown variables: *p*, λ_{HSA} and λ_{TA}. All coefficients are constant, while the voltage Φ_{TA} is varied quasi-statically. Parameters used in the calculations are listed in Table S1. The material properties μ and $J_{\lim}$ can be measured independently for both elastomers, and are assumed to be known, as well as the relative dielectric constant ε_r. The same holds for the volume of the connecting chamber V_C, the external pressure p_{atm}, the temperature *T*, and the unstretched balloon thicknesses *H*. The unstretched radii *R* (or corresponding volumes $V_0 = 4\pi R^3 / 3$) are inferred from the inflation/deflation experiments with a single balloon (see Figure S1, and the discussion after the Equation S8 and S9). In practice, *R* is an *equivalent spherical radius*, which is varied within physically admissible range for fitting. The amount of gas *N* is found from the *experimental* product on the left side of Equation S1. This product is constant within 0.3% everywhere during the cycle, which justifies our modelling approach.

It is instructive to analyze the behavior of the system in the plane of HSA volume and common pressure. The Equation $S2_{1\text{ or }3}$ defines a standard N-shaped single-balloon curve $p_{HSA}(V_{HSA})$.[12-14,35-37] This curve is shown as a solid blue line in Figure 4a. A similar dashed pink curve has smaller values of μ_{HSA} and $J_{\lim}$, to account for the inflation-deflation hysteresis in the material properties.

Equation S2$_{4\text{ or }5}$ defines an N-shaped curve $p_{TA}(V_{TA},\Phi_{TA})$, which depends also on the applied voltage Φ_{TA}. To present p_{TA} as a function of V_{HSA}, we find the latter from the conservation law in Equation S1 by substitution. This yields a parametric (V_{HSA},p) curve, with V_{TA} as a parameter along the curve:

$$(V_{HSA},p)=\left(\frac{NkT}{p_{atm}+p_{TA}(V_{TA},\Phi_{TA})}-V_C-V_{TA},p_{TA}(V_{TA},\Phi_{TA})\right) \quad \text{(S5)}$$

These curves are shown in Figure 4a and S2a for several voltages Φ_{TA}, using differently dotted black lines. The resulting curve on the right side of Equation S5 depends only on the properties of the TA balloon. We did not use the $p_{HSA}(V_{HSA})$ dependence at all. Thus, the black curves are independent of the blue—or pink—curve, which facilitates analysis and fitting. Their qualitative behavior can be understood as follows. For the used parameters, V_C is constant and the term $NkT/(p_{atm}+p_{TA})$ varies much less than V_{TA}. As a result, the dependence $p_{TA}(V_{TA})\approx p_{TA}(const-V_{HSA})$ reveals a *mirrored* N-shaped behavior in the (V_{HSA},p)-plane, depicted in Figure S2b over a larger pressure and volume scale. One can see, that this argumentation is *not exact* but very helpful for a deeper understanding.

The equilibrium of the system corresponds to the intersection of the blue—or pink—and black curves and moves along the former with changing voltage. The values of the voltages chosen for plotting in Figure 4a and S2a correspond to 0 V, snap-through, snap-back, and the maximum voltage. Snapping happens, when some of the common solutions disappear, i.e., when the blue—or pink—and the black curves become tangential to each other. The black curves indicate where the snapping *ends*, but the snapping path roughly follows the single HSA balloon curves (modified by dynamic effects). If the volume of the connecting chamber satisfies the condition $V_C >> V_{HSA}+V_{TA}$, the snap-through and snap-back curves are almost horizontal, with $p\approx const$. Larger voltages Φ_{TA} are required in this case.

The general analysis can be performed as follows: Substitute $p(\lambda_{HSA})$ from Equation S2$_3$ into the conservation law (Equation S1), and resolve it with respect to $V_{TA} \leftrightarrow \lambda_{TA}$. This gives the $\lambda_{TA}(\lambda_{HSA})$ dependence. Substitute $p(\lambda_{TA}, \Phi_{TA})$ from Equation S2$_5$ into the conservation law and resolve it with respect to $V_{HSA} \leftrightarrow \lambda_{HSA}$. This yields the $\lambda_{HSA}(\lambda_{TA}, \Phi_{TA})$ dependence. With this construction, $\lambda_{TA}(\lambda_{HSA})$ does not depend on TA *material* parameters, and $\lambda_{HSA}(\lambda_{TA}, \Phi_{TA})$ does not depend on HSA material values. The intersection of these two dependences in the $(\lambda_{HSA}, \lambda_{TA})$-plane for different values of the voltage Φ_{TA} defines the equilibrium of the system, and all variables can be expressed parametrically via either λ_{HSA} or λ_{TA}. The corresponding curves in the $(\lambda_{HSA}, \lambda_{TA})$ and (V_{HSA}, V_{TA})- planes are shown in Figure S3a and S3b.

The voltage Φ_{TA} is derived from the Equation S2$_5$, using the relation for the overpressure $p_{str}(\lambda_{TA})$ defined in Equation S2$_6$:

$$\frac{2\varepsilon_{TA}\Phi_{TA}^2\lambda_{TA}}{R_{TA}H_{TA}} = p_{str}(\lambda_{TA}) - p \tag{S6}$$

From here, using p a $p(\lambda_{HSA})$, and λ_{TA} a $\lambda_{TA}(\lambda_{HSA})$, as defined above, the equilibrium voltage can be expressed as a composite function of λ_{HSA}:

$$\Phi_{TA}(\lambda_{HSA}) = \sqrt{\frac{R_{TA}H_{TA}}{2\varepsilon_{TA}\lambda_{TA}(\lambda_{HSA})}(p_{str}[\lambda_{TA}(\lambda_{HSA})] - p(\lambda_{HSA}))} \tag{S7}$$

Equation S7 is used together with $V_{HSA} = V_{HSA,0}\lambda_{HSA}^3$ for the parametric dependence $(V_{HSA}, \Phi_{TA}) = (V_{HSA,0}\lambda_{HSA}^3, \Phi_{TA}(\lambda_{HSA}))$ shown in Figure 4b by the blue and pink curves. Dotted black lines correspond to the constant voltage of 0 V, snap-through, snap-back, and the maximum value.

Parameter estimation

The following observations elucidate the influence of different parameters on the system behavior. All parameters are listed in Table S1. The snap-through and snap-back happen near the maximal and minimal pressures of the $p_{HSA}(V_{HSA})$ curve. The *maximum* can be found in Neo-Hookean approximation:

$$\begin{aligned} & p \overset{1}{\approx} \frac{\mu H}{R} 2(\lambda^{-1} - \lambda^{-7}) \\ & \lambda_{\max} \overset{2}{\approx} 7^{1/6} \approx 1.38, \qquad V_{\max} \overset{3}{\approx} 7^{1/2} V_0 \approx 2.65 V_0, \; p_{\max} \overset{4}{\approx} 12 \times 7^{-7/6} \mu H / R \approx 1.24 \mu H / R \end{aligned} \tag{S8}$$

The *minimum* can be found keeping the leading powers of $\lambda >> 1$, because the next terms typically differ by a factor of λ^{-6}. For the Gent model in Equation S2₃, one can (numerically) recalculate $J_{\lim}, \lambda_{\lim}, V_{\lim}$ into each other using the relations $J_{\lim} = 2\lambda_{\lim}^2 + \lambda_{\lim}^{-4} - 3$, $V_{\lim} = V_0 \lambda_{\lim}^3$. This correspondence is listed in Table S2 (inflation data), and we can interchange these parameters at will. Good accuracy is provided by the following expressions.

$$\begin{aligned} & p \overset{1}{\approx} \frac{\mu H}{R} \frac{2\lambda^{-1}}{1 - (2\lambda^2 - 3)/(2\lambda_{\lim}^2 - 3)} \\ & \lambda_{\min} \overset{2}{\approx} \frac{\lambda_{\lim}}{\sqrt{3}} \approx 2.77, \qquad V_{\min} \overset{3}{\approx} \frac{\lambda_{\lim}^3}{3^{3/2}} V_0 = \frac{V_{\lim}}{3^{3/2}} \approx 21.2 V_0 = 0.19 V_{\lim}, \\ & p_{\min} \overset{4}{\approx} 3^{3/2} (1 - 3\lambda_{\lim}^{-2} / 2) \lambda_{\lim}^{-1} \frac{\mu H}{R} \approx 1.01 \frac{\mu H}{R} \end{aligned} \tag{S9}$$

The numerical values use the HSA data from Table S2. The Equations S8 and S9 are helpful for the fitting of the experimental data and the verification of material properties. The following ratios are useful:

$$\frac{V_{\min}}{V_{\max}} \approx \frac{\lambda_{\lim}^3}{7^{1/2} 3^{3/2}} \approx 8.02, \qquad \frac{p_{\max}}{p_{\min}} \approx \frac{12 \times 7^{-7/6}}{3^{3/2} (1 - 3\lambda_{\lim}^{-2}/2) \lambda_{\lim}^{-1}} \approx 1.22 \tag{S10}$$

The volume $V_{\max}$ yields the estimate for V_0 and *R*. The volume ratio $V_{\min} / V_{\max}$ estimates the maximal stretch $\lambda_{\lim}$ in Gent model. The pressure ratio $p_{\max} / p_{\min}$ provides a consistency

check for the material parameters, while $p_{\max}$ estimates the combination $\mu H / R$. If H is known, μ can be adjusted using this estimation and vice versa.

Figure S1a shows the fitting of the pressure-volume data for a *single* rubber HSA balloon with pronounced inflation/deflation hysteresis. This hysteresis is mainly due to the stretch-induced crystallization (SIC)[26,27,40] and viscoelasticity.[28] We do not discuss these effects here, but account for them phenomenologically, by using a smaller shear modulus μ_{HSA} and $J_{\lim}$ for the deflation stage, due to a higher crystalline fraction there. SIC softens the material with respect to the *overall* stretch, and shortens the chains in the *amorphous* fraction, as seen in the experimental hysteresis curves.[26,27,40] The μ_{HSA} value for deflation is slightly lower than in Figure 4, due to minor setup differences, such as a slightly larger maximal HSA extension (at which it was held for seconds), and 30 times slower pneumatic cycling in Figure S1a. These factors soften the elastomer and are reflected in a smaller μ_{HSA} value.

Figure S1b shows the fitting of the pressure-volume data for a *single* VHB TA balloon with compliant electrodes, but without applied voltage. As the TA balloon operates in a narrow interval of stretches and volumes, the inflation/deflation discrepancy is not crucial here. However, VHB is highly viscoelastic on a slow timescale preventing fast snap-through or snap-back.[15,16] VHB parameters change with time and the number of cycles. The first cycles differed significantly due to Mullins and Payne effect. After the 3rd cycle, which is fitted in Figure S1b, the differences became much less pronounced. The conditions for a single TA slightly deviated from the coupled case: No voltage on the TA, much slower pneumatic cycling, full stretching and relaxation cycle, as opposed to permanent high stretch with only moderate variations (shown in Figure 2d) and a shorter overall duration of the experiment. All these factors reduced long-term viscoelastic yielding, resulting in somewhat larger values for H and μ for the single TA membrane, which remained in the physically admissible range (see

Table S1). Fitting of the coupled system with these, or even larger *R*, *H*, μ values for the TA produced too flat snapping pressure behavior, and overly large HSA volumes as a result.

The Gent model provides a reasonable fit for the TA within its range of operation (200–400 cm^3). It is less suited for large VHB stretches, which are viscoelastic and rate dependent (right side of the blue and red curves in Figure S1b—rupture occurs at a volume of 1303.5 cm^3). The viscoelasticity of the TA also delays its elastic response to the applied voltage (as can be deduced from Figure 2c and 2f), leading to moderate discrepancies between theory and experiment, which can be seen near the maximum extension (zero voltage) point A in Figure 3b and 4b.

Approximation for the highly stretched TA

The parameters of the system are such that the TA remains highly stretched, with λ close to $\lambda_{\lim} >> 1$. Its volume *V* is close to $V_{\lim}$, and does not change much. In this situation, one can further simplify the approximation given by Equation S9$_1$, and express it in terms of volume *V*:

$$\frac{2\lambda^{-1}}{1-(2\lambda^2-3)/(2\lambda_{\lim}^2-3)} \approx \frac{1}{\lambda_{\lim}-\lambda} \approx \frac{3\lambda_{\lim}^{-1}V_{\lim}}{V_{\lim}-V} \tag{S11}$$

This gives the leading terms for the pressure of TA in Equation S2$_5$:

$$p_{TA} \approx \left(\frac{\mu H}{R}\frac{3\lambda_{\lim}^{-1}V_{\lim}}{V_{\lim}-V} - \frac{2\varepsilon\Phi^2\lambda_{\lim}}{RH} \right)_{TA} \tag{S12}$$

This result depends on $V_{\lim}$ rather than on V_0 (although both are related) and the difference of *V* and $V_{\lim}$. A value for $V_{\lim}$ can be deduced from the logarithmic derivative of Equation S12 for $\Phi = 0$, which results in:

$$V_{\lim} = \frac{\Delta(pV)}{\Delta p} \tag{S13}$$

$V_{\lim}$ can be estimated from Equation S13, using two or several adjacent experimental data points. The same expression holds with V a λ, but λ is not directly measured in the experiments.

Influence of the voltage

To understand the influence of the voltage, we note that snap-through occurs near the $(V_{\max}, p_{\max})$ values of the HSA balloon, see Equation S8$_{3,4}$, and snap-back near $(V_{\min}, p_{\min})$ given by Equation S9$_{3,4}$. In the lowest-order approximation for a very large connecting chamber the volume of the TA does not change significantly, and the black curves in Figure 4a become almost horizontal. Then, the change in the *elastic* HSA pressure between the snapping points should be matched by the *changes* in the *electrostatic* pressure in the strongly stretched TA:

$$\begin{aligned} & p_{\max} - p_{\min} \approx \left(C \frac{\mu H}{R} \right)_{HSA} \approx \left(\frac{2\varepsilon \lambda_{\lim}}{HR} \right)_{TA} (\Phi^2_{TA,back} - \Phi^2_{TA,through}) \\ & C \approx 12 \times 7^{-7/6} - 3^{3/2}(1 - 3\lambda_{\lim}^{-2}/2)\lambda_{\lim}^{-1} \approx 0.23 \end{aligned} \tag{S14}$$

This relation elucidates main *trends* in the system parameter dependences. However, it cannot be used for *quantitative* predictions, because for the current setup, the slope of the black curves is significant, and the snap-back deviates substantially from the $(V_{\min}, p_{\min})$ point (Fig. 4a). The situation is further complicated by the SIC hysteresis in the HSA and viscoelastic effects in the TA.

In optimal operating conditions the snap-through occurs at minimal voltage, $\Phi_{through} \approx 0$. $p_{\max}$ of the HSA should be matched by the pressure of the strongly stretched TA. This helps to choose its parameters, such as thickness *H*, or pre-stretch (i.e., effective *R* and V_0). Then, the minimal possible snap-back voltage can be estimated from the Equation S14.

$$\Phi_{\substack{back \\ \text{minimal}}} \approx \left(\frac{HR}{2\varepsilon\lambda_{\text{lim}}}\right)_{TA}^{1/2} \left(C\frac{\mu H}{R}\right)_{HSA}^{1/2} \tag{S15}$$

For our numbers this gives 2.4 [kV] using inflation values (2.58 [kV] accounting for inflation/deflation hysteresis), which lies in the realistic range. Used conditions are not far from optimal, because $\Phi^2_{through}[\text{kV}] \approx \underbrace{1.8^2}_{3.24} = \underbrace{3.9^2}_{15.21} \approx \Phi^2_{back}$. The volume range of actuation can be only moderately increased without drawbacks. A significantly larger actuation magnitude requires bigger actuators, which can be readily designed using the guidelines described in the Equation S8, S9, S14 and S15.

Estimation of the force for sorting applications

The force acting on the drinking cup in Figure 1c can be deduced from the Video S1 (see Supporting Video). After the contact with the high-speed actuator the cup's velocity is about 1.8 m/s. For a cup of 5 g, this corresponds to a momentum of about 9 x 10-3 kg m/s. With a contact time of 36 ms (9 frames of the high-speed video at 250 fps) this suggests a (normal) force of 0.25 N acting on the cup. The (maximal) gripping force depends on the particular sorting geometry and can be estimated from the overpressure in the HSA, which is around 25 mbar (Figure 3). Considering the current setup, contact areas of up to 40 cm^2 are feasible, providing a (normal) force around 10 N. Even when assuming a rather low friction coefficient of 0.1, objects of 100 g mass can be readily handled. Such forces can be easily tolerated by a human. The punch of the actuator was tested by bare hand and does not present any hazard in operation.

References

[35] K. Volokh, *Mechanics of soft materials*, Springer, New York, USA **2016**, p. 155.

[36] A. F. Bower, *Applied mechanics of solids*, CRC Press, Boca Raton, USA **2010**, p. 794.

[37] G. A. Holzapfel, *Nonlinear solid mechanics*, Wiley, Chichester, USA **2000**, p 455.

[38] C. Keplinger, M. Kaltenbrunner, N. Arnold, S. Bauer*, Proc. Natl. Acad. Sci.* **2010**, *107*, 4505.

[39] E. M. Mockensturm, N. Goulbourne, *Int. J. Non. Lin. Mech.* **2006**, *41*, 388.

[40] A. Gros, B. Huneau, E. Verron, M. Tosaka, *J. Mech. Phys. Solids* **2019**, *125*, 164.

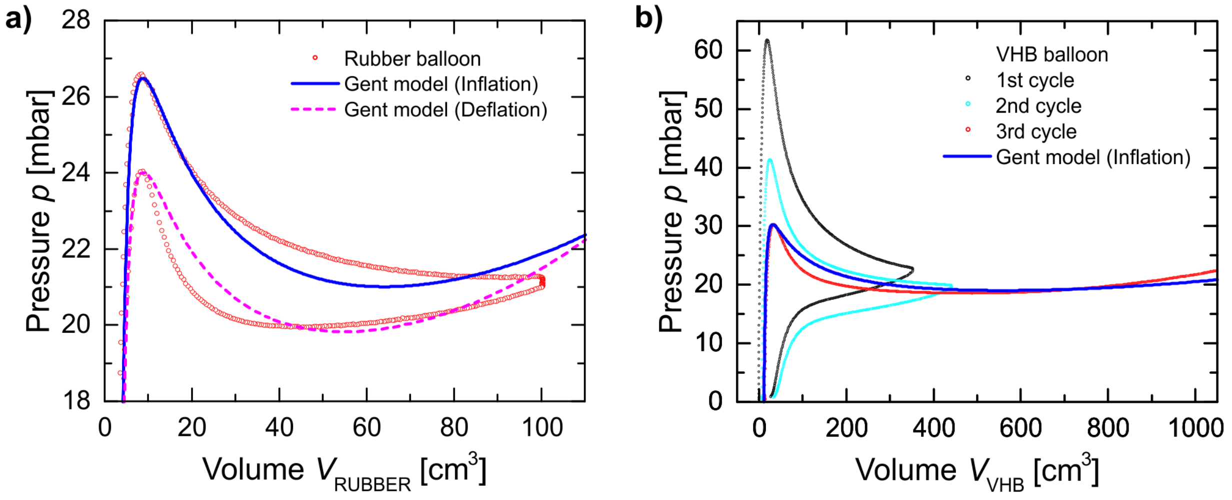

Figure S1. a) Fitting of the pressure-volume data of the inflation and deflation of the rubber membrane (HSA alone) using the Gent model. b) Inflation of the VHB balloon (TA alone). The 3rd cycle is fitted, the modulus in the cycling experiments is lower due to accumulated viscoelastic effects. All parameters are listed in Table S1.

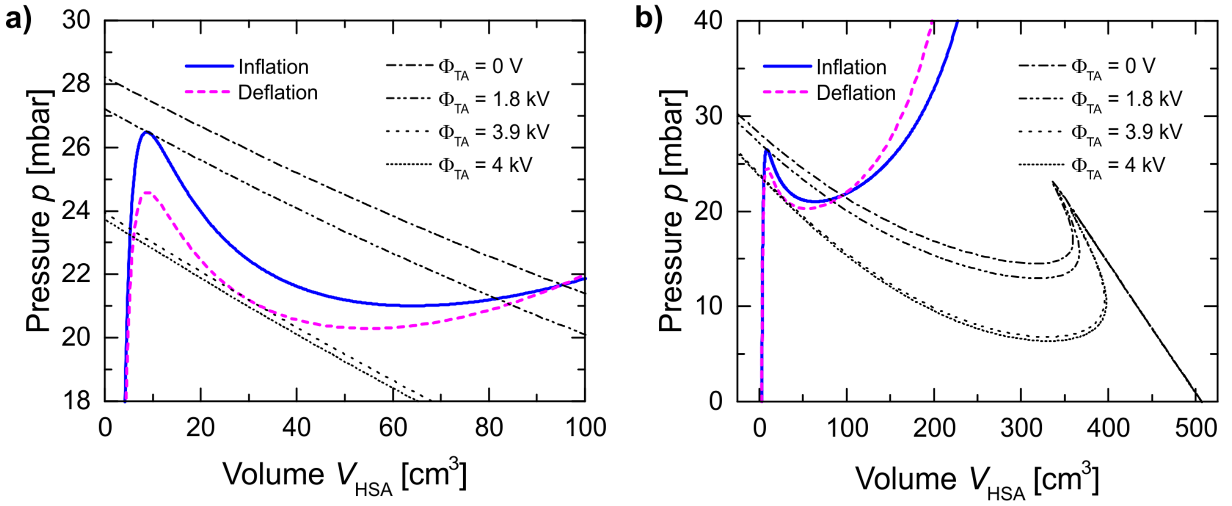

Figure S2. a) The Figure 4a from the main text is reproduced here in simplified form for convenience. b) The same dependence as in (a), over a wider range of volumes and pressures. The black dotted curves are based on the single-balloon $p_{\mathrm{TA}}(V_{\mathrm{TA}})$ dependence combined with the conservation law (Equation S1), and demonstrate a *mirrored* N-shaped dependence. The system operates in the range of large stretches λ_{TA}.

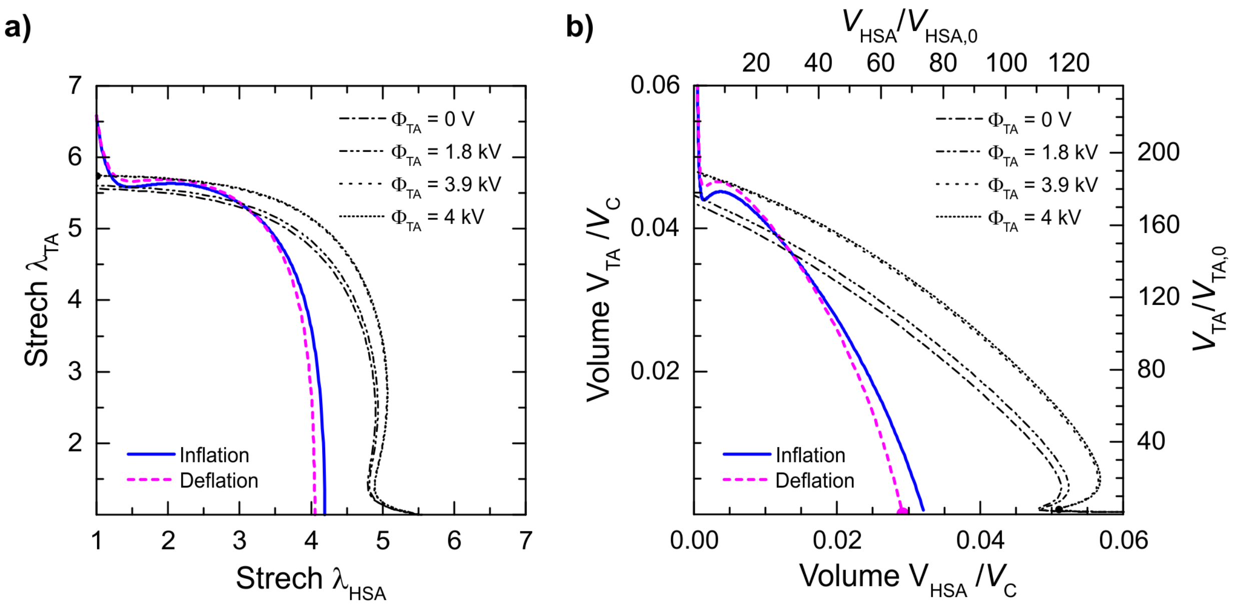

Figure S3. a) Interrelation between the stretches, which follows from the ideal gas law (Equation S1). Solid blue and dashed pink curves $\lambda_{TA}(\lambda_{HSA})$ are obtained using the substitution of $p(\lambda_{HSA})$. Dotted black curves $\lambda_{HSA}(\lambda_{TA},\Phi_{TA})$ are obtained using the substitution of $p(\lambda_{TA},\Phi_{TA})$. All parameters are the same as in Figure 4 or S2. b) Dependences from Figure S3a (recalculated into the balloon volumes) normalized to the volume of the connecting chamber. A slightly different range of stretches is shown, to make the volume scales the same for both axes.

Table S1. Parameters used in the calculations

Balloon actuators	μ [kPa]	J_{lim}	H [cm]	R [cm]	V_0 [cm^3]	ε_r
Coupled TA	16	77	0.086	0.75	1.77	4.7
TA alone [a)]	33.7	77	0.1	1.4	11.5	
Coupled HSA (infl.; defl.) [b)]	374; 346	43; 38.5	50×10^{-4}	0.9	3.05	
HSA alone (infl.; defl.) [a,b)]	374; 338	43; 38.5	50×10^{-4}	0.9	3.05	
Common parameters	p_{atm} [kPa]	NkT [J]	T [K]	N [mole]	V_C [cm^3]	
	101.325	760.8	293.15	0.312	7000	

[a)] Experiment with only a single balloon of the corresponding material; [b)] Different values for inflation and deflation account for the material hysteresis

Table S2. Secondary calculation parameters

Balloon actuators	λ_{lim}	R_{lim} [cm]	V_{lim} [cm^3]	$\Phi_{through}$ [kV]	Φ_{back} [kV]
TA	6.32	4.74	447.1	1.8 (1.825) [a)]	3.9 (3.975) [a)]
HSA [b)]	4.8	4.32	336.8		

[a)] Experimental; [b)] Inflation parameters are used

Supplementary Videos

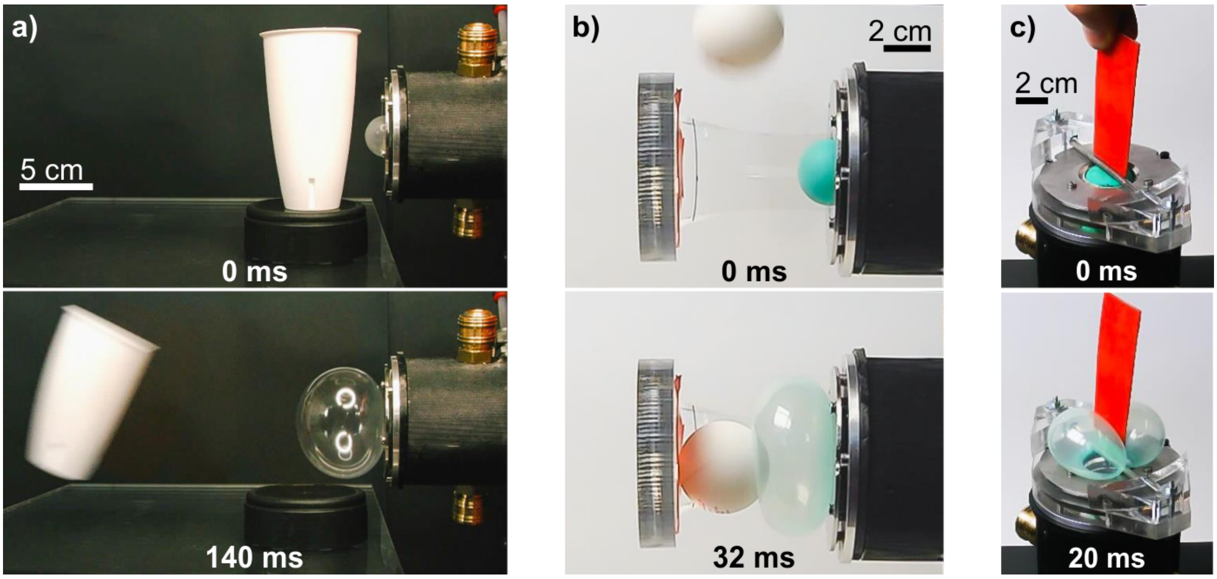

Video S1. Snapshots from the Video *'Prototypes utilizing snap-through instability'* demonstrating possible applications in real-time and slow-motion: (a) '*snap-sorting'*, (b) '*snap-catching'* and (c) '*snap-gripping'*.

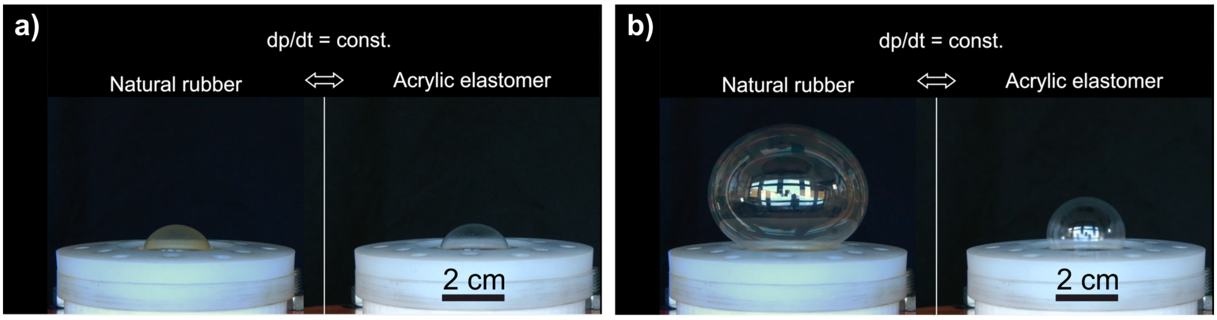

Video S2. Still images of the Video *'Comparison of natural rubber and the acrylic elastomer VHB'* showing the temporal response.

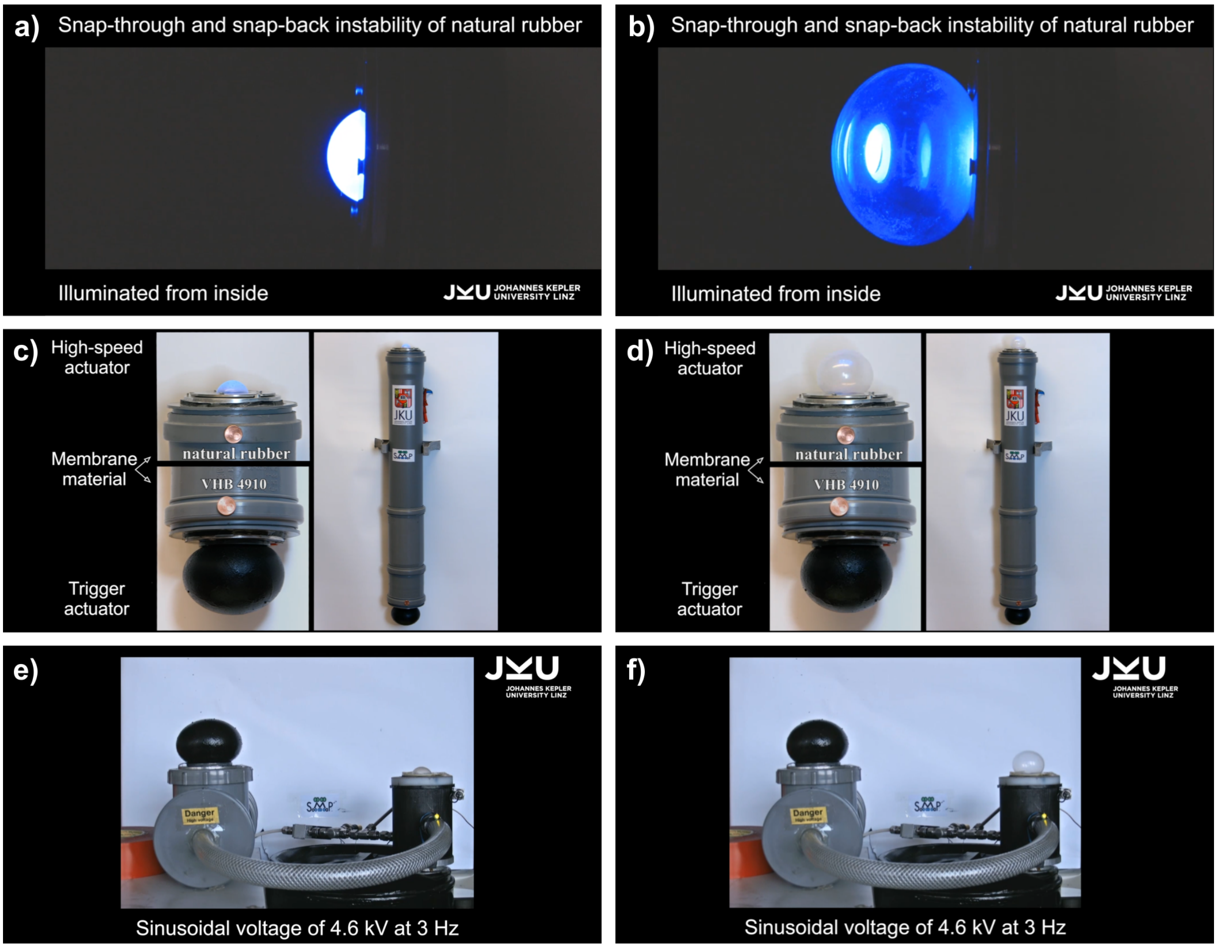

Video S3. Still images of the Video *'High-speed voltage-triggered soft actuator harnessing instability'* illustrating the fast response due to the snap-through and snap-back instability of natural rubber in (a) and (b), the principal set-up in (c) and (d), as well as the cyclic deformation by applying sinusoidal voltage (4 kV at several frequencies) to the trigger actuator in (d) and (f).
